# Supplementary material for: Clinically distinct metabotypes of pediatric MASLD identified through unsupervised clustering of NASH CRN data
Source: Nat Commun. 2026 Feb 24;17:3107. doi: 10.1038/s41467-026-69735-z (PMC13039967; doi:10.1038/s41467-026-69735-z)

Figure S1. Comparison of Observed and Permuted Average Silhouette Widths Across Different Numbers of Clusters. Average silhouette widths for both observed (black) and permuted (red) data are displayed across different numbers of clusters (k), ranging from 2 to 10. The highest observed silhouette width occurs at k = 3, suggesting this is the optimal number of clusters. Permuted silhouette widths remain consistently lower than observed values, indicating stronger clustering performance in the actual data compared to random permutations. A decline in silhouette width is observed as k increases beyond 3, highlighting diminishing cluster separation with more clusters.

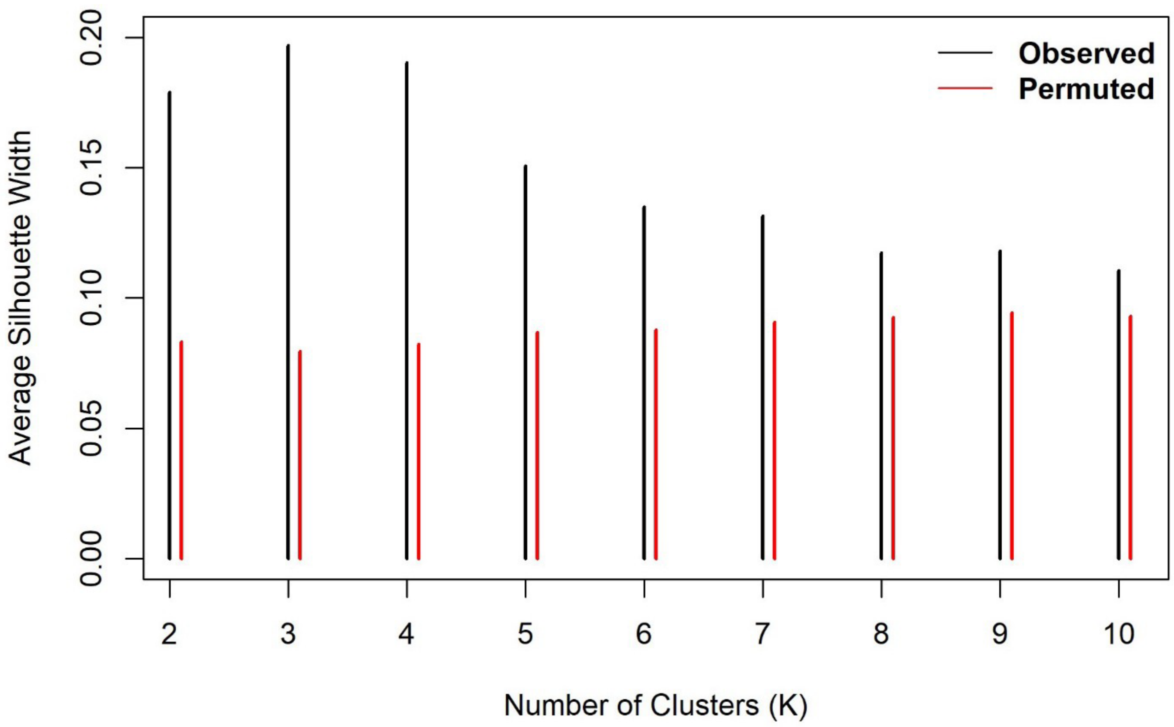

Figure S2. K-means clustering visualizations stratified by sex. Clustering of participants (n = 514 children with MASLD) was performed on scaled clinical variables, with participants colored by sex. Dimensionality reduction was achieved using Principal Component Analysis (PCA) to project the data onto the first two principal components for visualization. The k-means algorithm was configured with three clusters (k = 3), using 25 random starts and a maximum of 1000 iterations. The metabotypes are labeled Cardiometabolic (CM, blue), Early-mild (EM, green), and Inflammatory-fibrotic (IF, orange).

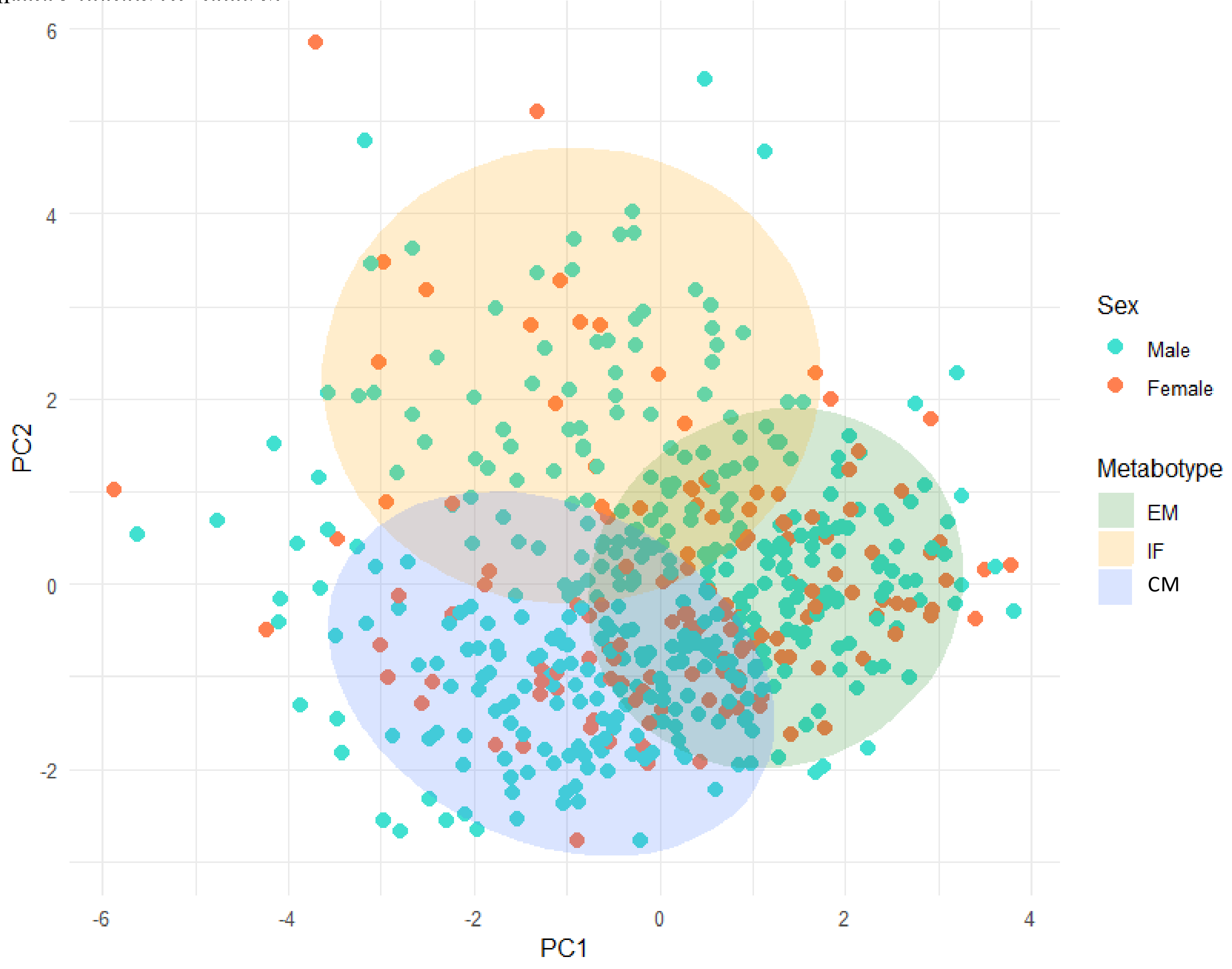

Figure S3. Principal Component Analysis (PCA) and Partial Least Squares Discriminant Analysis (PLS-DA) of metabolomics data obtained from the HILIC+ mode. (A) PCA scores plot, with PC1 explaining 6.9% of the variance and PC2 explaining 4.3%. **(B)** PLS-DA scores plot, with Component 1 explaining 3.5% of the variation and Component 2 explaining 4%. The ellipses represent 95% confidence intervals for each group. The metabotypes are labeled Cardiometabolic (CM, red), Early-mild (EM, green), and Inflammatory-fibrotic (IF, blue). **(C)** Top 3 HILIC+ annotations identified by one-way ANOVA. Pairwise comparisons were calculated using Mann-Whitney U tests, with p-values denoting significant differences ($p < 0.05$). Black horizontal lines indicate group medians.

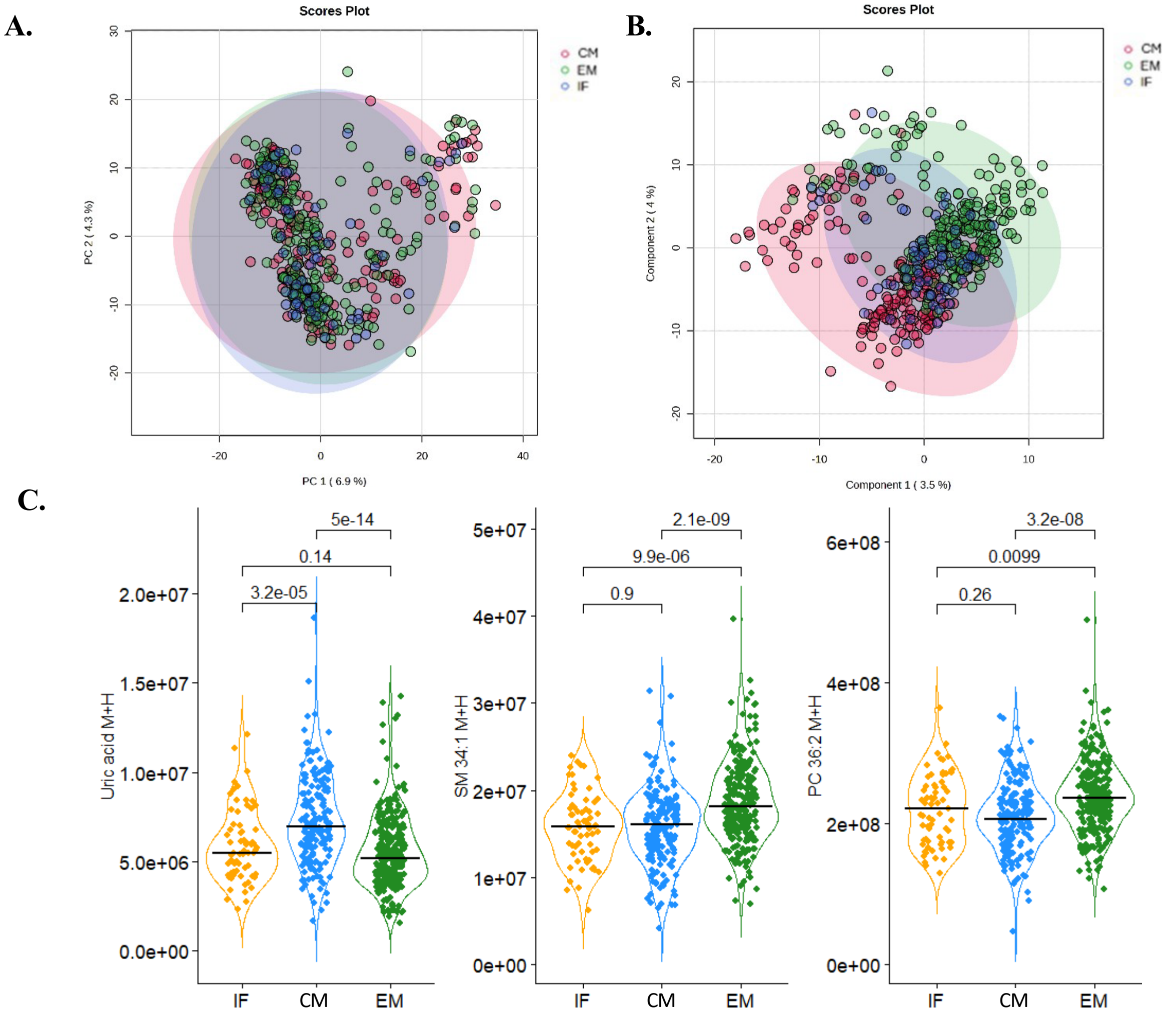

Figure S4. Principal Component Analysis (PCA) and Partial Least Squares Discriminant Analysis (PLS-DA) of metabolomics data obtained from the C18- mode. (A) PCA scores plot, with PC1 accounting for 6% of the total variance and PC2 for 2.6% **(B)** PLS-DA scores plot, with Component 1 explaining 3% of the variation and Component 2 explaining 2.6%. The ellipses represent the 95% confidence intervals for each group. The metabotypes are labeled Cardiometabolic (CM, red), Early-mild (EM, green), and Inflammatory-fibrotic (IF, blue). **(C)** Top 3 C18- annotations identified by one-way ANOVA. Pairwise comparisons were calculated using Mann-Whitney U tests, with p-values denoting significant differences ($p < 0.05$). Black horizontal lines indicate group medians.

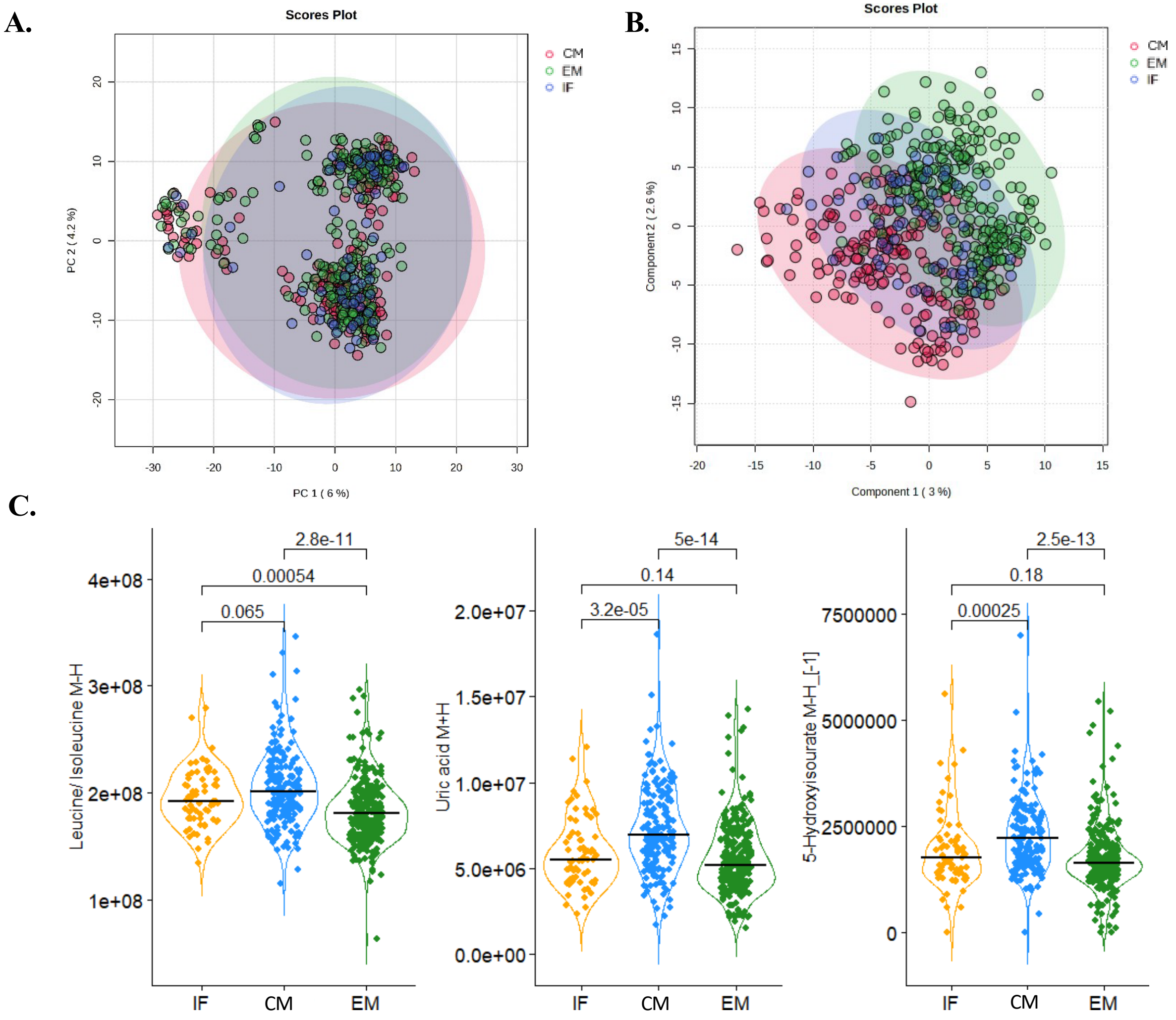

Figure S5. Tryptophan metabolism across metabotypes. (A) Heatmap showing differences in significant metabolites of tryptophan metabolism across metabotypes IF, CM, and EM from HILIC+ mode. **(B)** Violin plots illustrating pairwise comparisons of metabolite features across metabotypes, calculated using Mann-Whitney U tests, with p-values denoting significant differences ($p < 0.05$). Black horizontal lines indicate group medians, highlighting central tendencies within each metabotype.

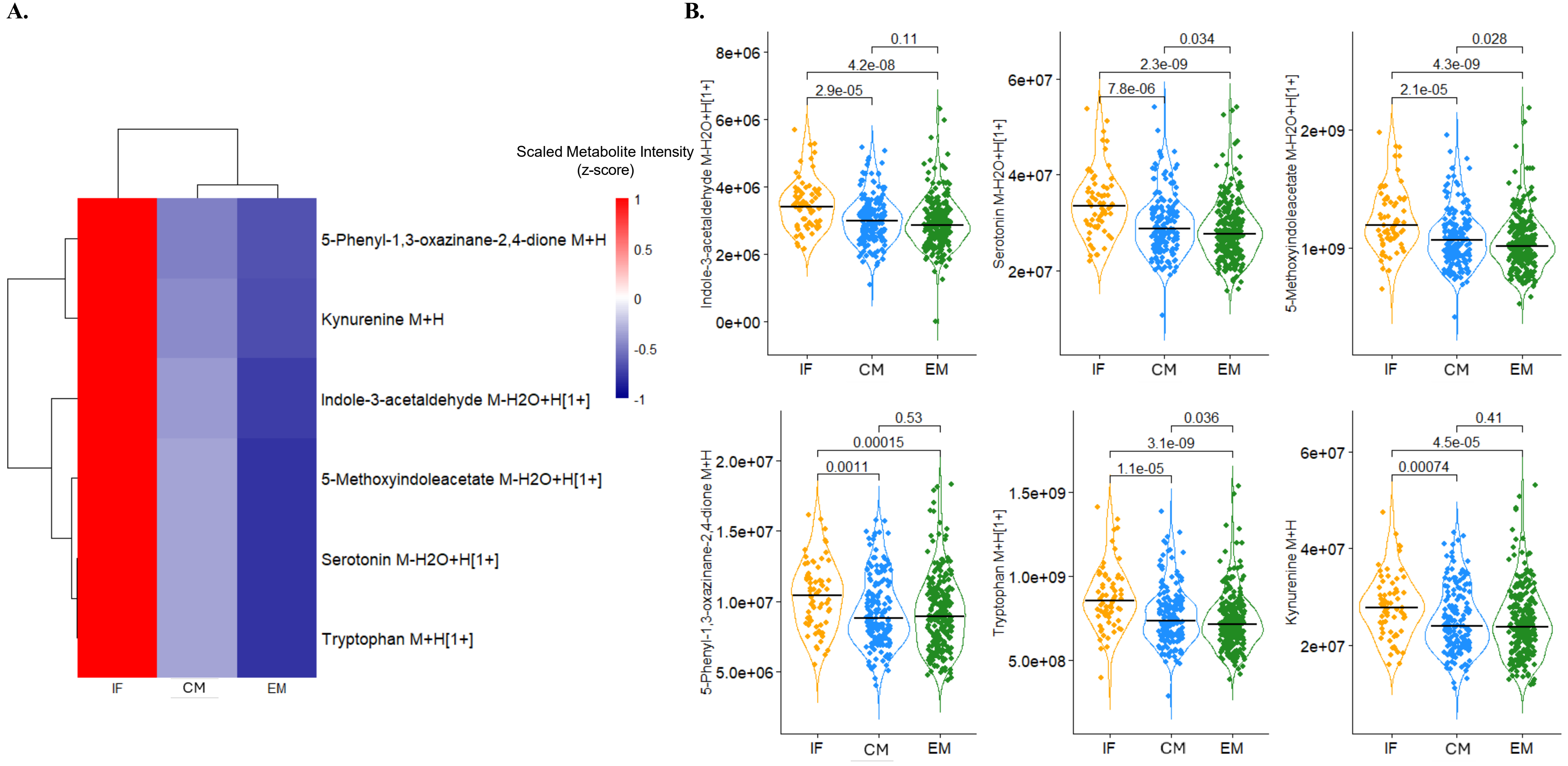

Figure S6. Pantothenate and CoA biosynthesis across metabotypes. (A) Heatmap showing differences in significant metabolites of pantothenate and CoA biosynthesis across metabotypes IF, CM, and EM from C18- mode. **(B)** Violin plots illustrating pairwise comparisons of metabolite features across metabotypes, calculated using Mann-Whitney U tests, with p-values denoting significant differences ($p < 0.05$). Black horizontal lines indicate group medians, highlighting central tendencies within each metabotype.

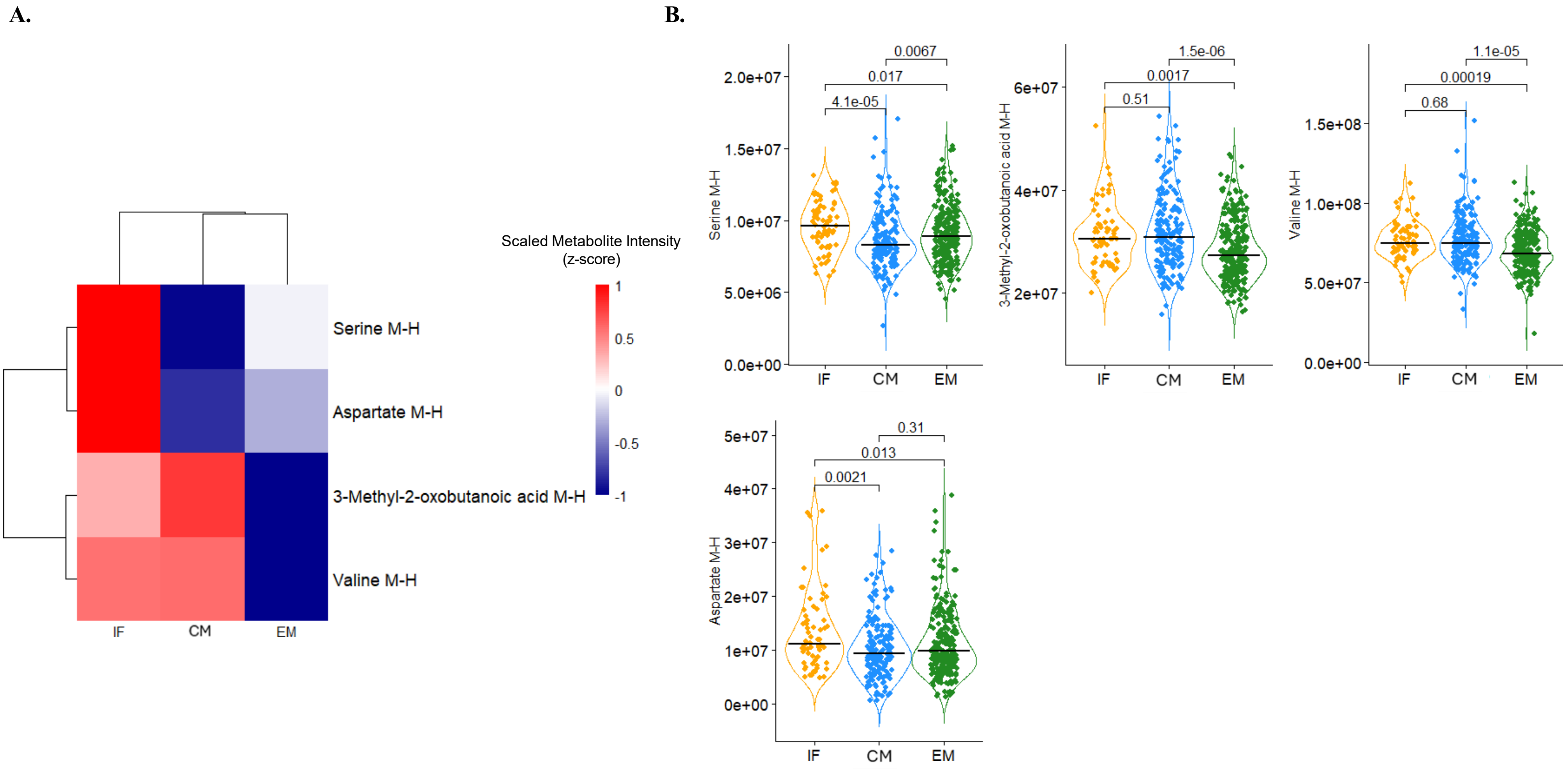

Figure S7. Branched-chain amino acid (BCAA) degradation across metabotypes. (A) Heatmap showing differences in significant metabolites of BCAA degradation across metabotypes IF, CM, and EM from C18- mode. **(B)** Violin plots illustrating pairwise comparisons of metabolite features across metabotypes, calculated using Mann-Whitney U tests, with p-values denoting significant differences ($p < 0.05$). Black horizontal lines indicate group medians, highlighting central tendencies within each metabotype.

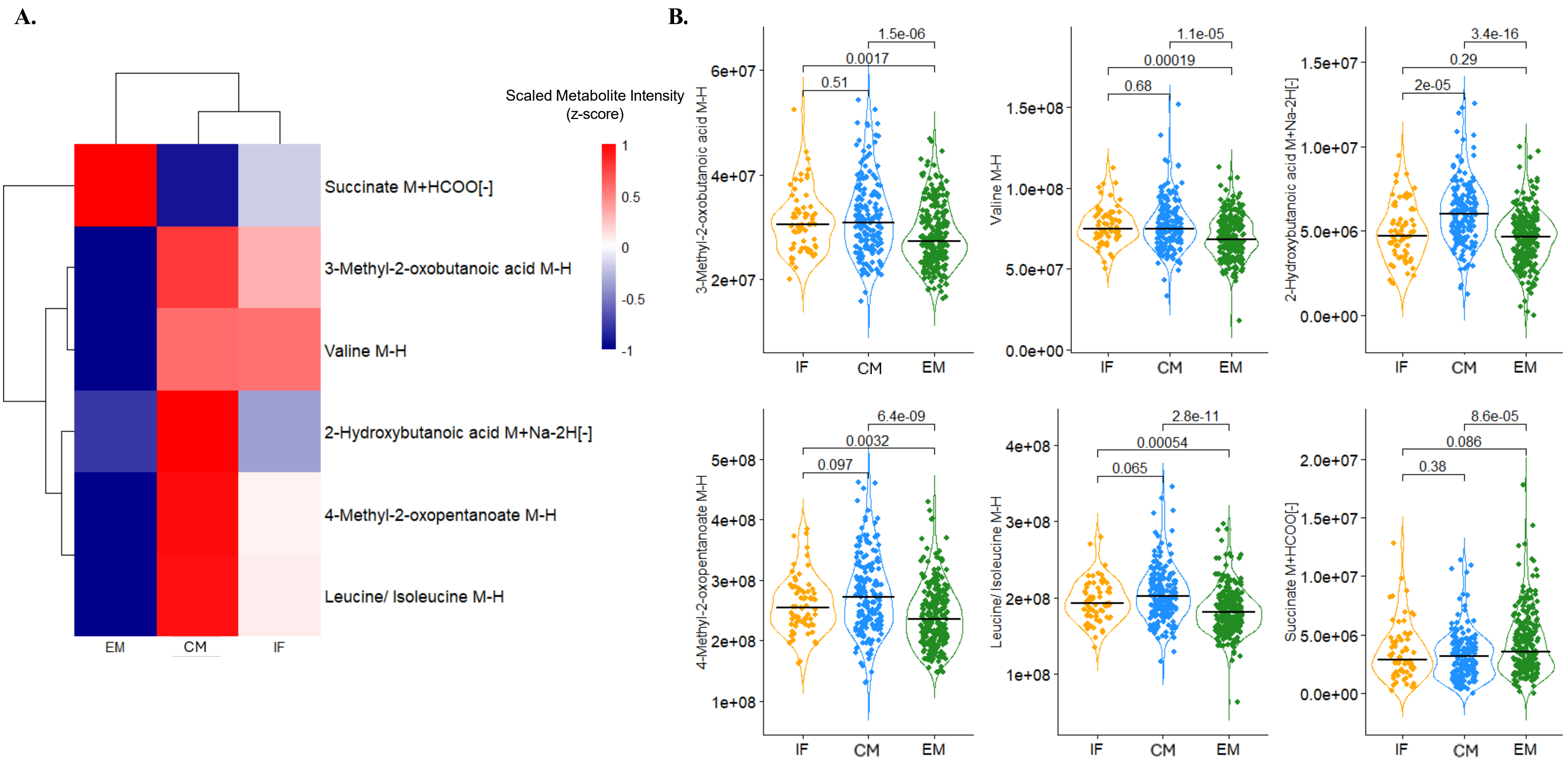

Figure S8. Butanoate metabolism across metabotypes. (A) Heatmap showing differences in significant metabolites of butanoate metabolism across metabotypes IF, CM, and EM from C18-mode. **(B)** Violin plots illustrating pairwise comparisons of metabolite features across metabotypes, calculated using Mann-Whitney U tests, with p-values denoting significant differences ($p < 0.05$). Black horizontal lines indicate group medians, highlighting central tendencies within each metabotype.

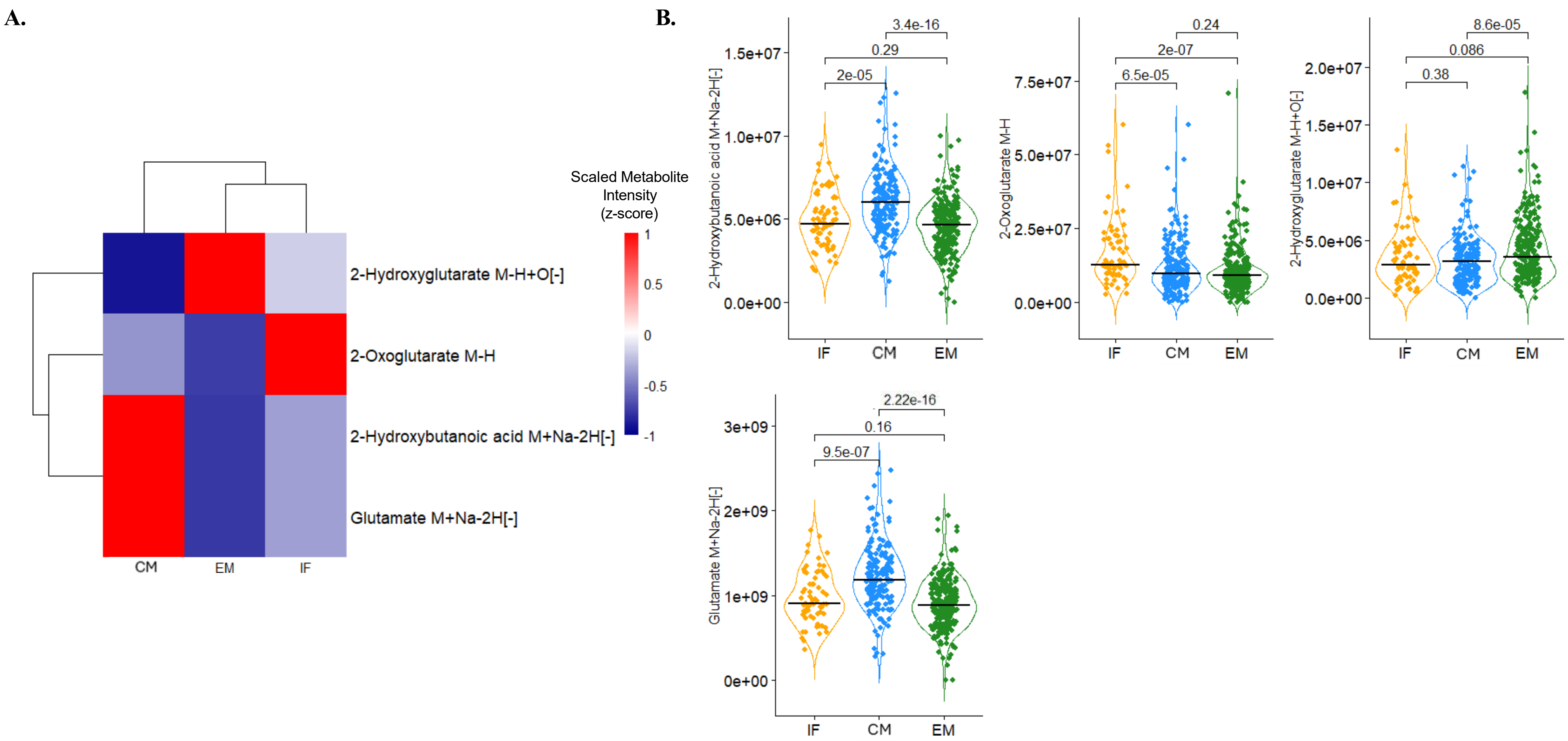

Figure S9. Propanoate metabolism across metabotypes. (A) Heatmap showing differences in significant metabolites of propanoate metabolism across metabotypes IF, CM, and EM from C18- mode. **(B)** Violin plots illustrating pairwise comparisons of metabolite features across metabotypes, calculated using Mann-Whitney U tests, with p-values denoting significant differences ($p < 0.05$). Black horizontal lines indicate group medians, highlighting central tendencies within each metabotype.

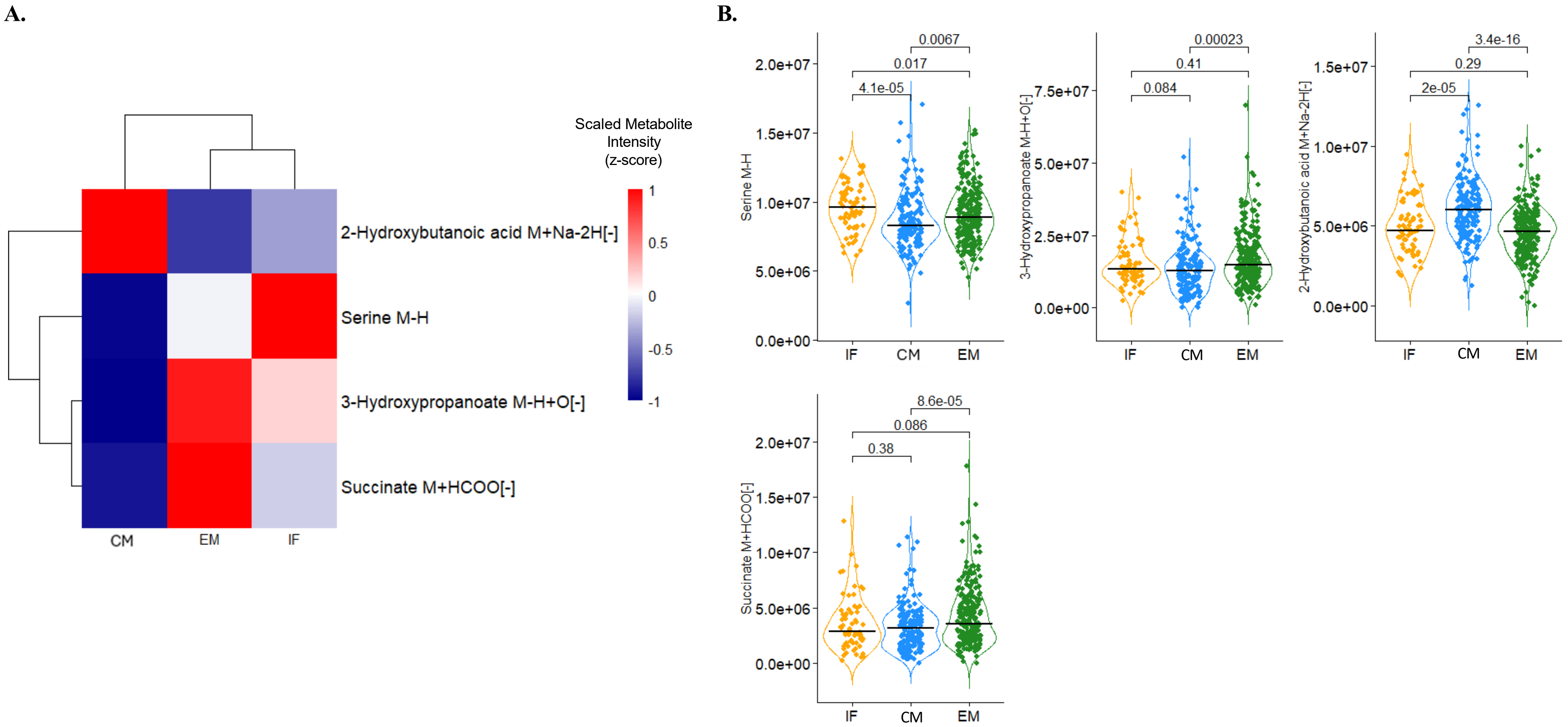

Figure S10. Purine metabolism across metabotypes. (A) Heatmap showing differences in significant metabolites of purine metabolism across metabotypes IF, CM, and EM from HILIC+ mode. **(B)** Violin plots illustrating pairwise comparisons of metabolite features across metabotypes, calculated using Mann-Whitney U tests, with p-values denoting significant differences ($p < 0.05$). Black horizontal lines indicate group medians, highlighting central tendencies within each metabotype.

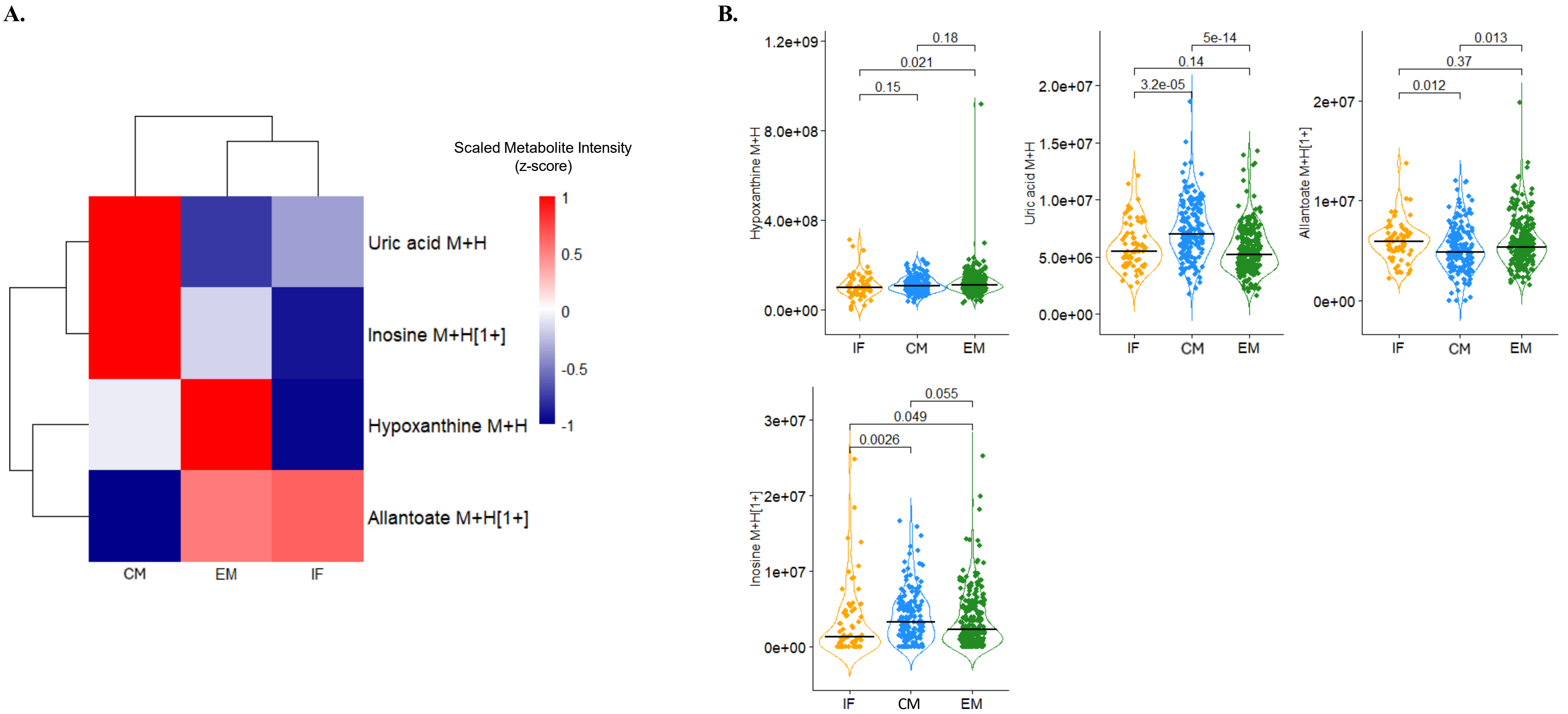

Figure S11. Differences in enriched pathway metabolites between EM and CM metabotypes. (A) Heatmap showing differences in metabolites of significantly enriched pathways between the EM and CM metabotypes, with a color gradient indicating higher levels (red) and lower levels (blue).

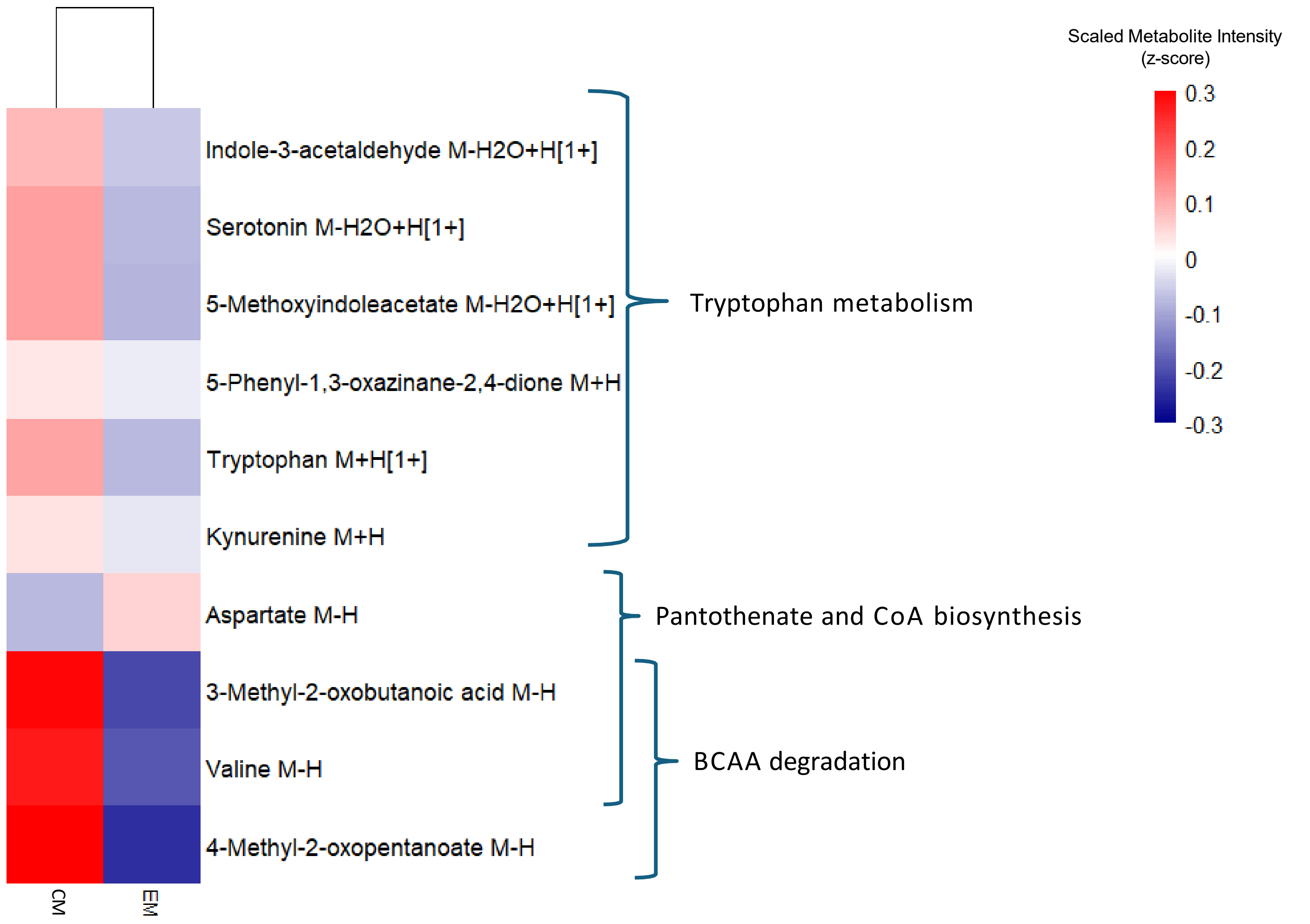

Figure S11. Differences in enriched pathway metabolites between EM and CM metabotypes. (B) Violin plots illustrating pairwise comparisons of metabolite features between CM and EM, calculated using Mann-Whitney U tests, with p-values denoting significant differences ($p < 0.05$). Black horizontal lines indicate group medians, highlighting central tendencies within each metabotype.

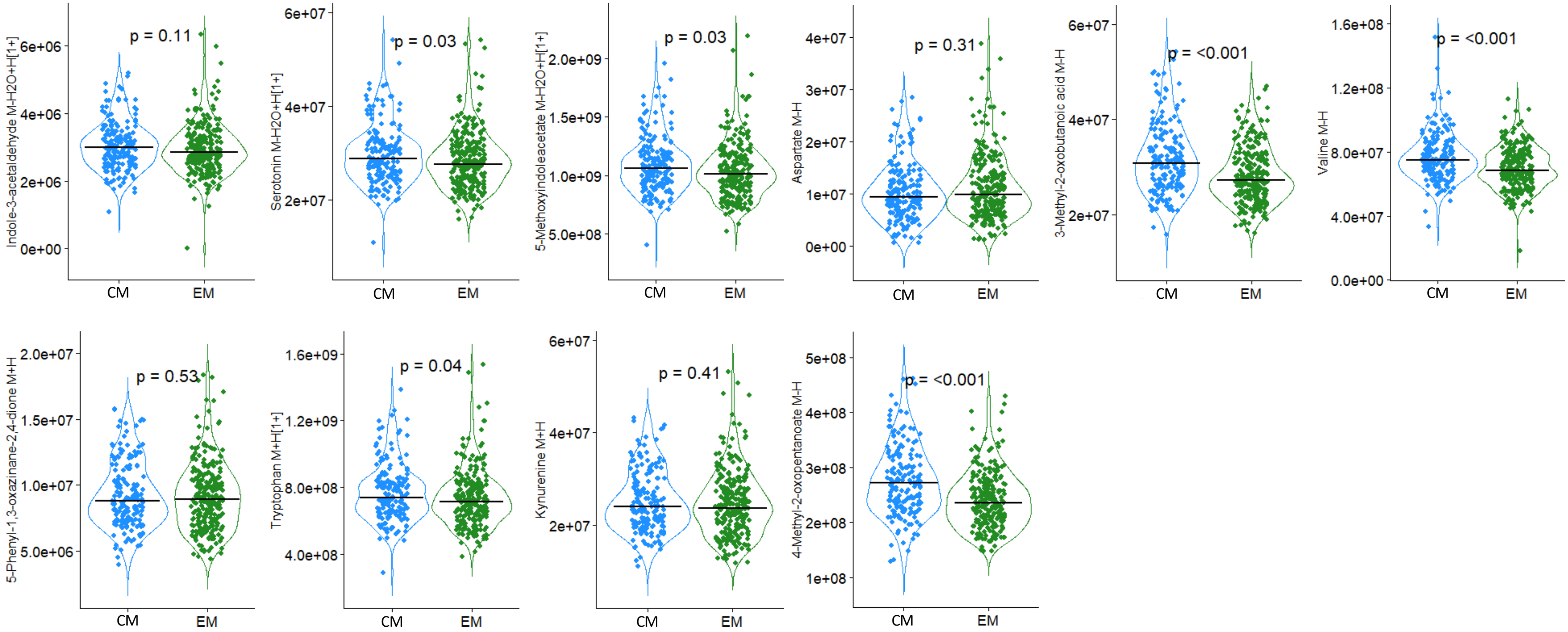

Figure S12. Differences in enriched pathway metabolites between EM and IF metabotypes. (A) Heatmap showing differences in metabolites of significantly enriched pathways between the EM and IF metabotypes, with a color gradient indicating higher levels (red) and lower levels (blue).

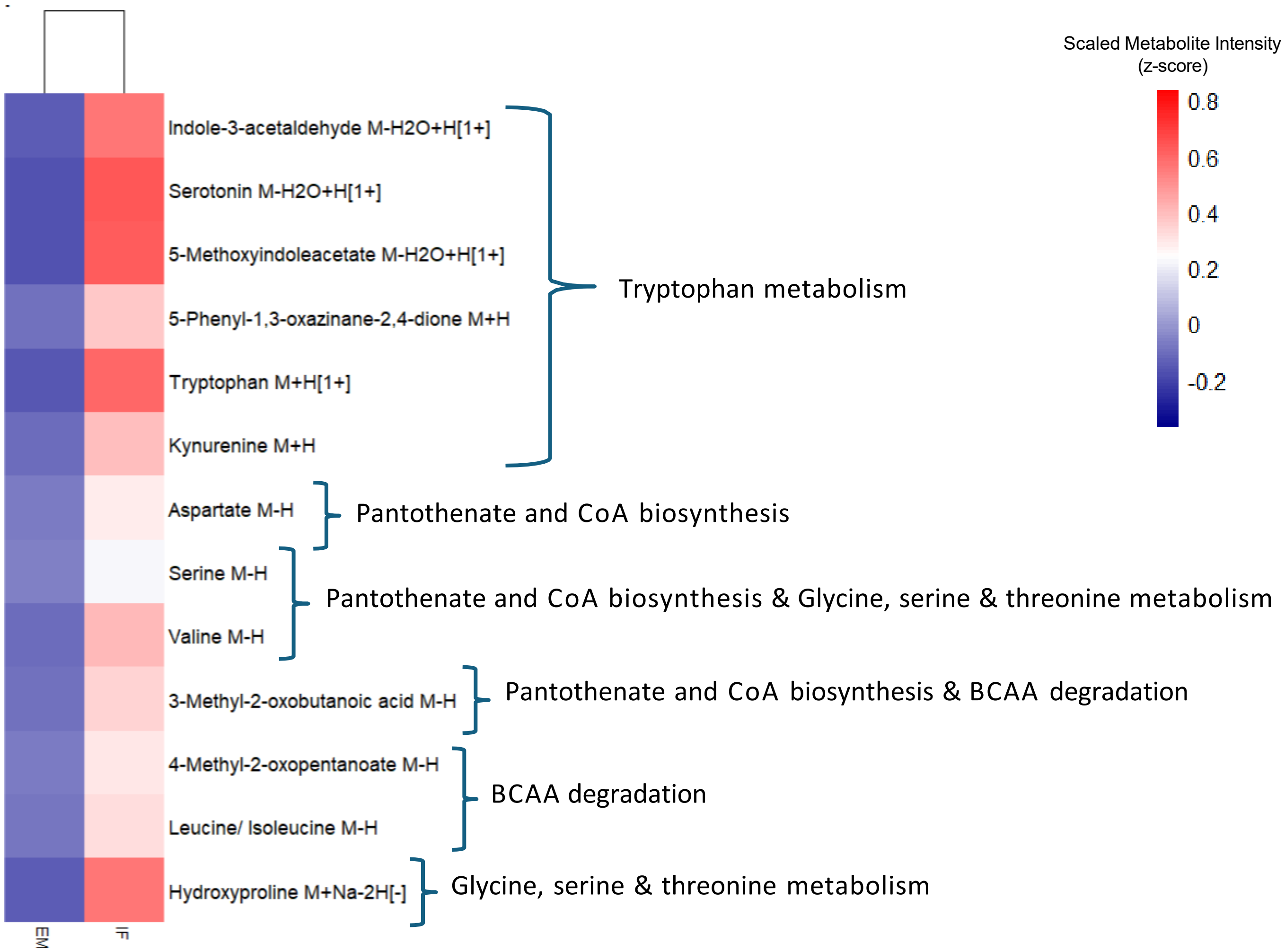

Figure S12. Differences in enriched pathway metabolites between EM and IF metabotypes. (B) Violin plots illustrating pairwise comparisons of metabolite features between IF and EM, calculated using Mann-Whitney U tests, with p-values denoting significant differences ($p < 0.05$). Black horizontal lines indicate group medians, highlighting central tendencies within each metabotype.

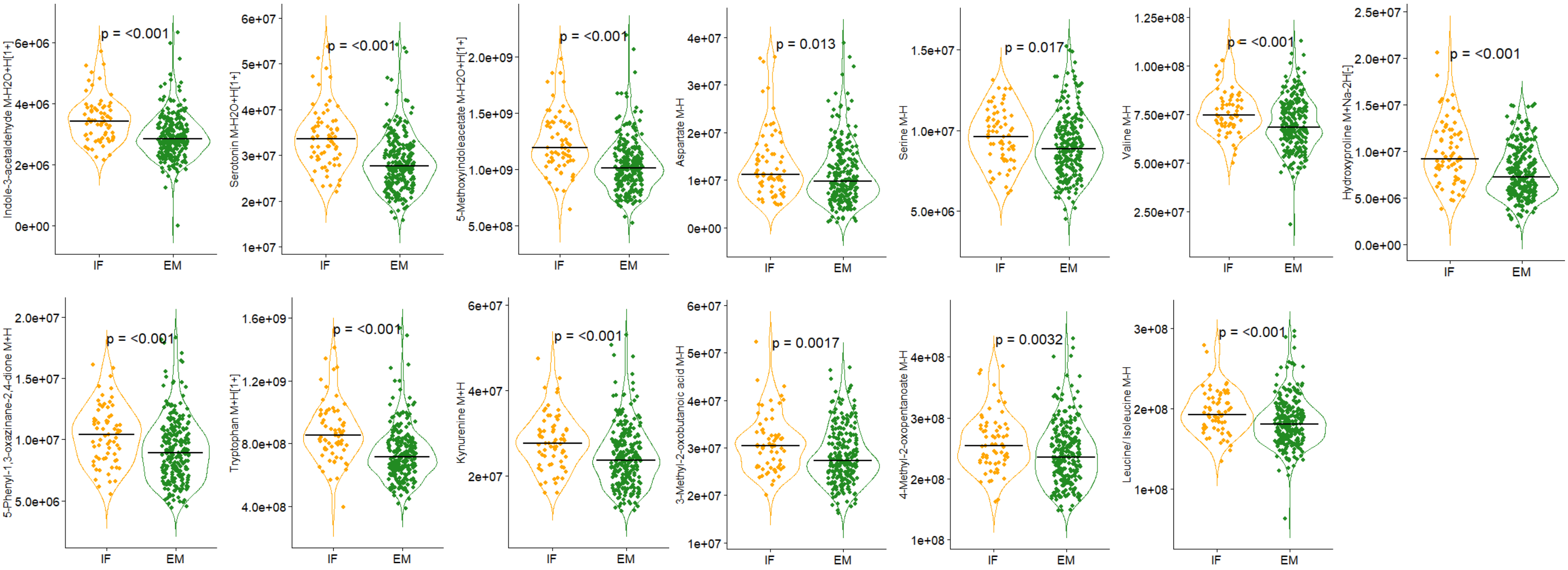

Figure S13. Differences in enriched pathway metabolites between IF and CM metabotypes. (A) Heatmap showing differences in metabolites of significantly enriched pathways between the IF and CM metabotypes, with a color gradient indicating higher levels (red) and lower levels (blue).

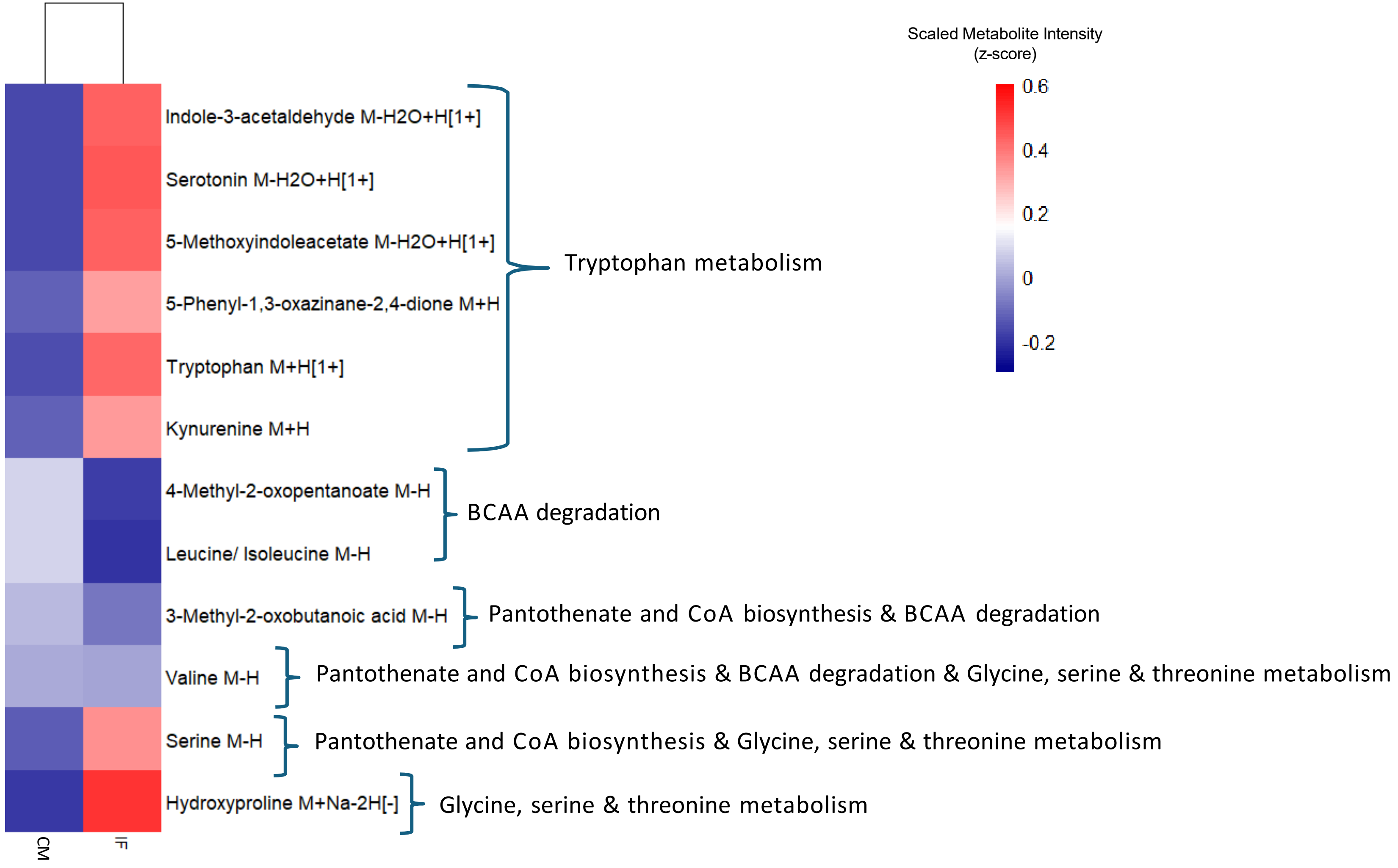

Figure S13. Differences in enriched pathway metabolites between IF and CM metabotypes. (B) Violin plots illustrating pairwise comparisons of metabolite features between IF and CM, calculated using Mann-Whitney U tests, with p-values denoting significant differences ($p < 0.05$). Black horizontal lines indicate group medians, highlighting central tendencies within each metabotype.

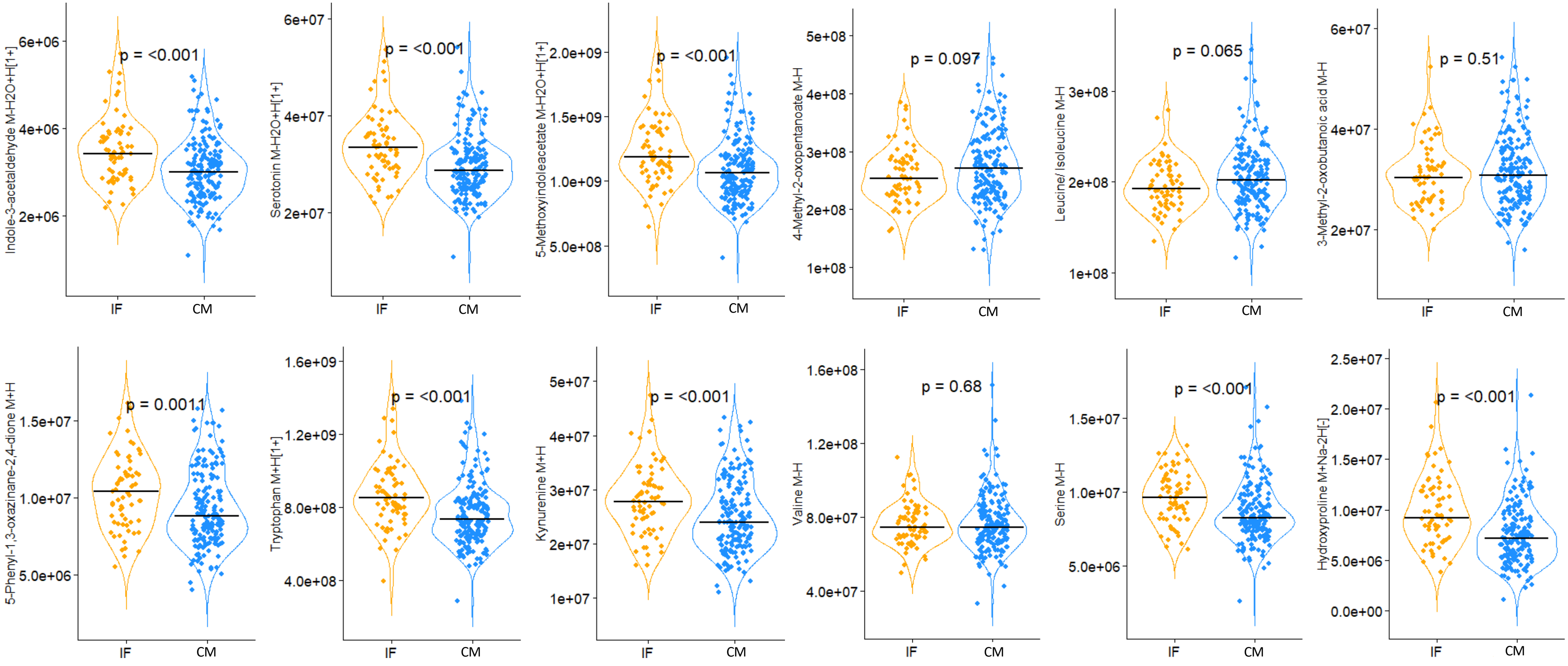

Figure S14. Sample selection for the NASH CRN cohort. Flow chart illustrating the sample selection process for the NASH CRN cohort.

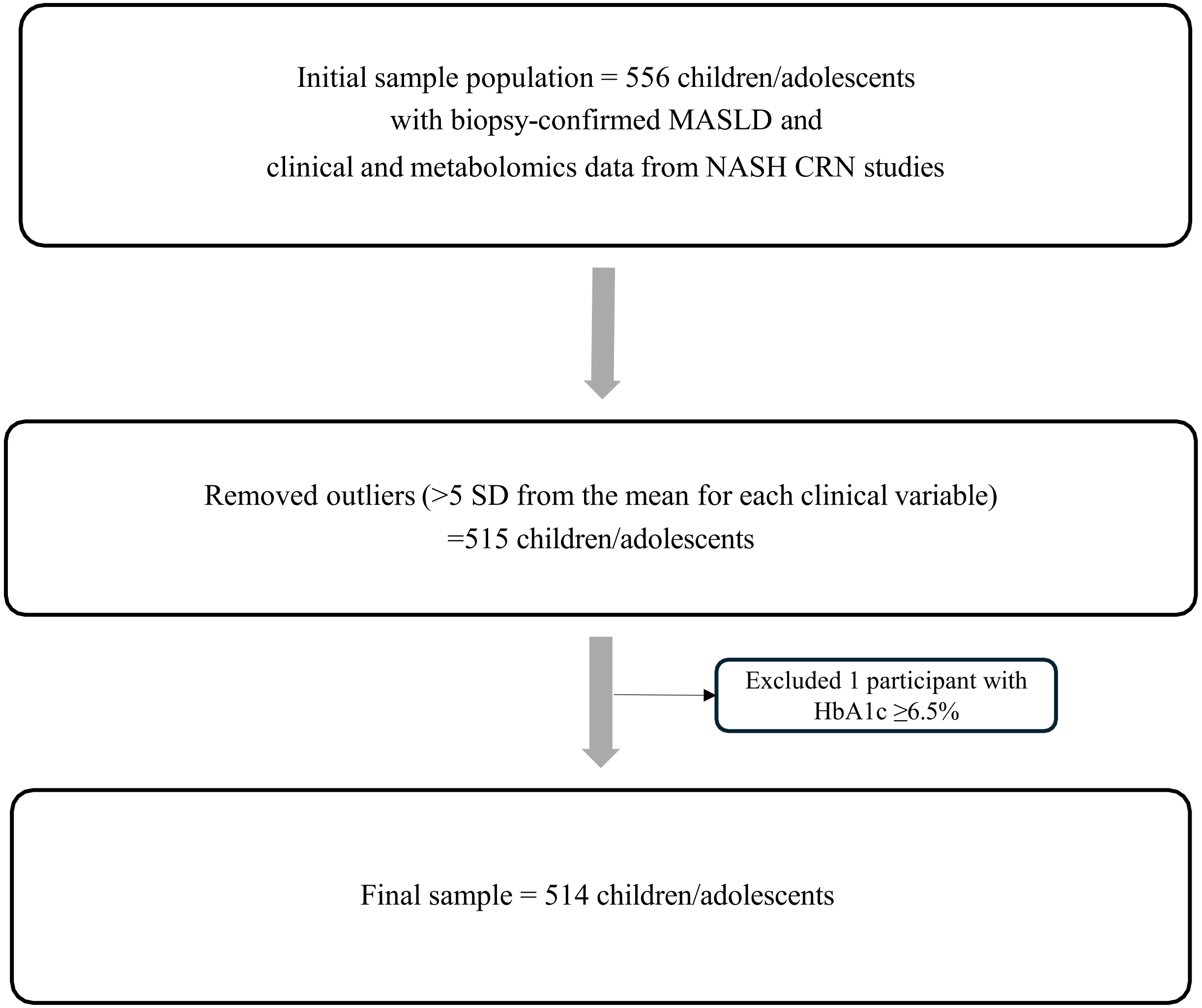

Figure S15. Silhouette width plot. The optimal number of clusters was determined using the silhouette method. The plot displays the average silhouette width as a function of the number of clusters (k), ranging from 1 to 10. A peak silhouette width is observed at k = 3, indicating that this is the optimal number of clusters. After k = 3, the silhouette width gradually decreases, suggesting diminishing cluster quality with the addition of more clusters.

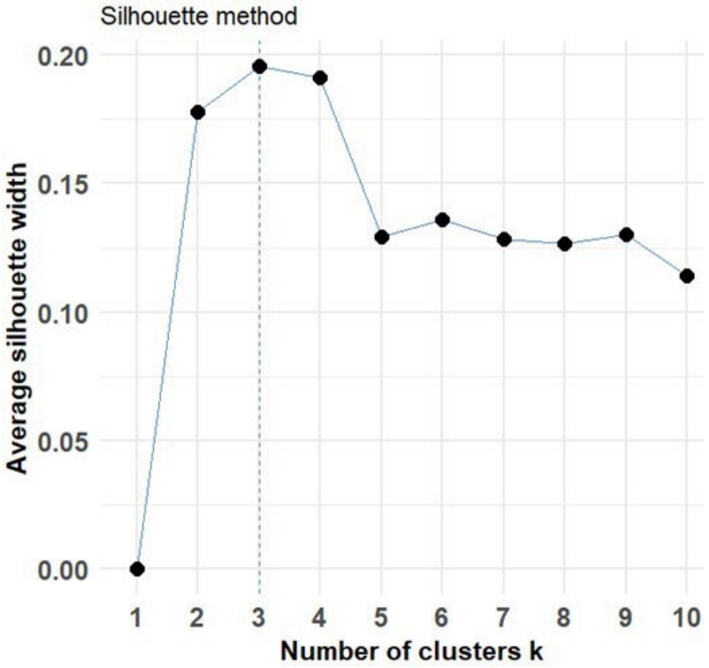

Figure S16. Number of clusters recommended by *NbClust* Indices. Bar plot illustrating the number of clusters recommended by different indices from the *NbClust* analysis. The analysis used the k- means method with Euclidean distance testing cluster numbers from 2 to 10 (min.nc = 2, max.nc = 10) and included all available indices (index = "all"). The majority of indices (26) recommended 3 clusters as optimal.

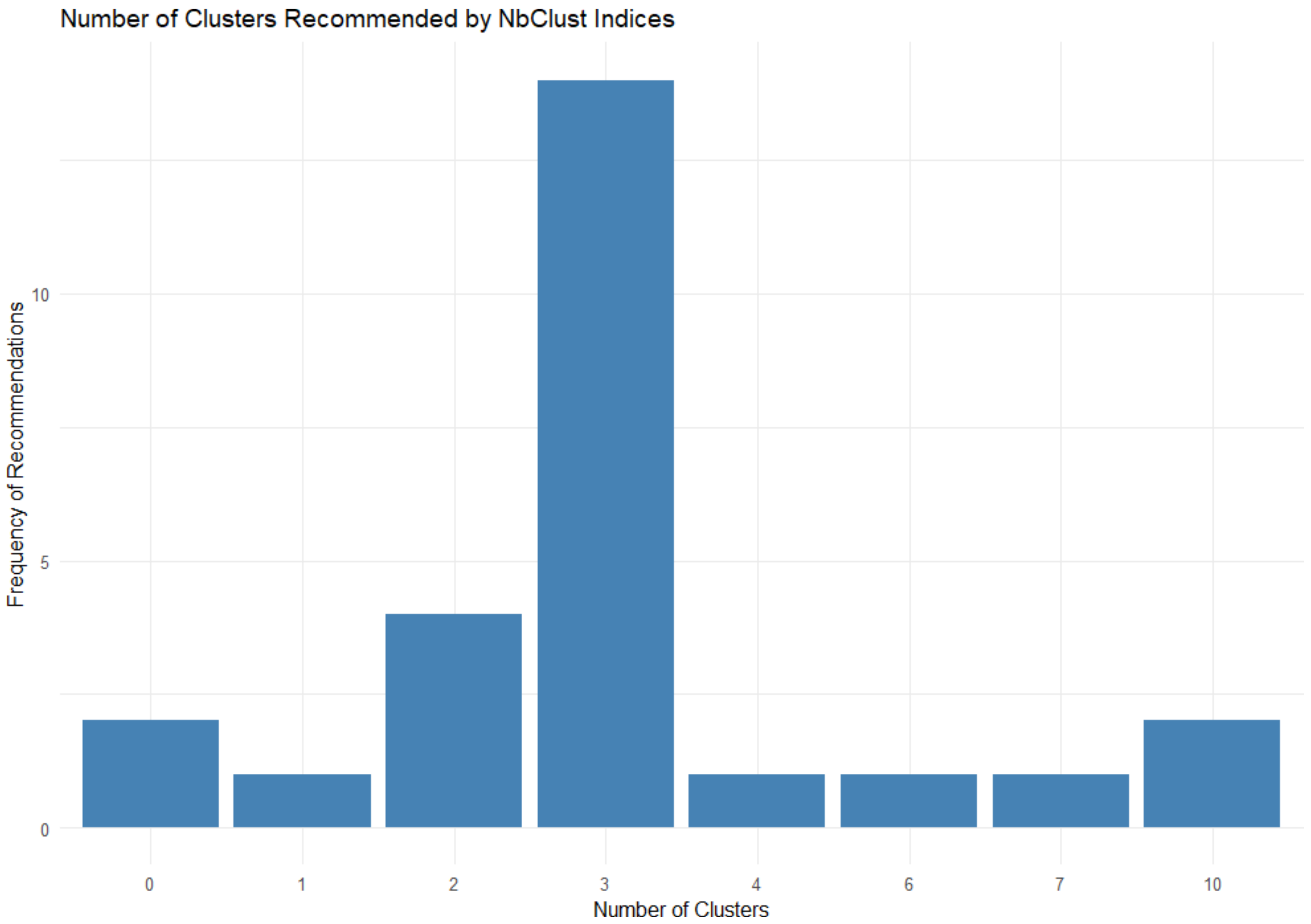

Supplement: Supplementary file 1 — Supplementary Information [file 41467_2026_69735_MOESM1_ESM.pdf]
