# Supplementary material for: Clinically distinct metabotypes of pediatric MASLD identified through unsupervised clustering of NASH CRN data
Source: Nat Commun. 2026 Feb 24;17:3107. doi: 10.1038/s41467-026-69735-z (PMC13039967; doi:10.1038/s41467-026-69735-z)
Supplement: Supplementary file 2 — Description of Additional Supplementary Files [file 41467_2026_69735_MOESM2_ESM.pdf]

Description of Additional Supplementary Files

Title: Supplementary Data 1. Clinical and demographic characteristics by NASH CRN study.
Description: Clinical, histologic, and demographic characteristics of 514 children and adolescents (5-18 years) with biopsy-proven MASLD, stratified by NASH CRN Studies. Participant characteristics are presented for the three NASH CRN study cohorts: DB1, DB2, and TONIC. Continuous variables reported as median (interquartile range) and categorical variables as frequencies (percentages). Comparisons across the three NASH CRN studies were performed using two-sided Kruskal–Wallis tests for continuous variables and two-sided chi-squared or Fisher's exact tests for categorical variables, as appropriate. Statistically significant differences ($p < 0.05$) are highlighted in bold.

Title: Supplementary Data 2. Differential HILIC+ metabolites across metabotypes.
Description: Metabolites extracted by hydrophilic interaction chromatography and detected by positive electrospray ionization that significantly differed between metabotypes (IF, EM, CM). Mass spectral features are reported by mass-to-charge ratio (m/z) and retention time (RT, s). Statistical significance is provided with p-value and false discovery rate (FDR). Tukey's HSD results indicate specific pairwise differences between metabotypes. Signal intensities (minimum, mean, and maximum) illustrate the range of feature levels across samples. The top 25 most significant features identified by one-way ANOVA across the three metabotypes were annotated using the internal reference library and xMSannotator. Schymanski Level Confidence (SLC) is provided for each annotation. Metabolites confirmed by coelution and MS/MS against authentic standards are designated as Level 1, following Schymanski classification criteria (see Liu et al., 2020, Analytical Chemistry for details).

Title: Supplementary Data 3. Differential C18- metabolites across metabotypes.
Description: Metabolites extracted by C18 chromatography and detected by negative electrospray ionization that significantly differed between metabotypes (IF, EM, CM). Mass spectral features are reported by mass-to-charge ratio (m/z) and retention time (RT, s). Statistical significance is provided with p-value and false discovery rate (FDR). Tukey's HSD results indicate specific pairwise differences between metabotypes. Signal intensities (minimum, mean, and maximum) illustrate the range of feature levels across samples. The top 25 most significant features identified by one-way ANOVA across the three metabotypes were annotated using the internal reference library and xMSannotator. Schymanski Level Confidence (SLC) is provided for each annotation. Metabolites confirmed by coelution and MS/MS against authentic standards are designated as Level 1, following Schymanski classification criteria (see Liu et al., 2020, Analytical Chemistry for details).

Title: Supplementary Data 4. Metabolite annotation confidence for pathway enrichment analyses.
Description: Metabolite identification confidence for pathway enrichment analysis results provided in Figure 2. Pathways significantly enriched between the metabotypes from HILIC+ and C18– mode were identified using Mummichog version 2.0 with KEGG human pathways. For each pathway, features are reported with their corresponding metabolite candidates, formulas, adducts, and Schymanski Level Confidence (SLC). Annotation was performed using the internal reference library and xMSannotator.

Metabolites confirmed via coelution and MS/MS relative to authentic standards are reported as SLC Level 1. SLC Level 5 was assigned in two situations: (i) features with an xMSannotator confidence score of 0, reflecting exact mass matches only, and (ii) features mapped by mummichog to a candidate metabolite (e.g., 3-methyl-2-oxobutanoic acid, 4-methyl-2-oxopentanoate) that could not be corroborated by either xMSannotator or the internal reference library. See Liu et al., 2020 (Analytical Chemistry) for details on SLC criteria.

Title: Supplementary Data 5. Network centrality measures across pediatric MASLD metabotypes.
Description: (A) Centrality scores of metabolite features across three distinct pediatric MASLD metabotypes: IF, EM, and CM. Mass spectral features are reported by mass-to-charge ratio (m/z) and retention time (RT, s). Centrality values indicate the relative importance of each feature within the network for each metabotype. The table includes columns for each metabotype as well as comparisons between the groups (e.g., EM_vs_CM, IF_vs_CM, IF_vs_EM) to highlight differences in feature centrality among the metabotypes. (B)Centrality scores of clinical variables and fibrosis stage across the 3 pediatric MASLD metabotypes: IF, EM, and CM. Centrality values indicate the relative importance of each feature within the network for each metabotype. The table includes columns for each metabotype as well as comparisons between the groups (e.g., EM_vs_CM, IF_vs_CM, IF_vs_EM) to highlight differences in feature centrality among the metabotypes.

Title: Supplementary Data 6. Metabolite confidence for xMWAS-based pathway enrichment.
Description: Metabolite identification confidence for pathway enrichment analysis based on xMWAS centrality differences (≥0.025) between the metabotypes (see Figure S15A). Metabolic pathways significantly enriched between the metabotypes from HILIC+ and C18- mode with metabolites mapped to Kyoto Encyclopedia of Genes and Genomes (KEGG) human pathways using Mummichog version 2.0. For each pathway, metabolites are provided with Schymanski Level Confidence (SLC), formula, and adduct forms matched by Mummichog. Metabolites confirmed via coelution and MS/MS relative to authentic standards are designated as Level 1; please see Liu et al 2020 Anal. Chem for more specific information.

Title: Supplementary Data 7. Network correlations between clinical variables and tryptophan metabolites.
Description: xMWAS network correlations between clinical variables, fibrosis stage, and tryptophan metabolites.

Title: Supplementary Data 8. Metabolite correlations with fibrosis stage.
Description: Significant correlations between fibrosis stage and metabolite features from HILIC+ and C18- modes. Statistical significance was set at $p<0.05$. Spearman rank correlations were calculated between each metabolite feature and fibrosis score using the rcorr function in the Hmisc package. All statistical tests were two-sided, and p-values were not adjusted for multiple comparisons. The top 20 HILIC+ and C18- metabolite features were annotated using the internal reference library and xMSannotator. Schymanski Level Confidence (SLC) is provided for each annotation. Metabolites

confirmed by coelution and MS/MS against authentic standards are designated as Level 1, following Schymanski classification criteria (see Liu et al., 2020, Analytical Chemistry for details). Abbreviations: PA, phosphatidic acid; PC, phosphatidylcholine; PE, phosphatidylethanolamine; PI, phosphatidylinositol. O-denotes an ether-linked species.

Title: Supplementary Data 9. HILIC+ features used for pathway enrichment analysis.
Description: HILIC+ dataset used for pathway enrichment analysis in MetaboAnalyst (Mummichog v2.0). Significant features (one-way ANOVA, FDR-adjusted $p<0.05$) were used as input, with enrichment evaluated by permutation testing against all quality-filtered m/z features. Pathways were considered enriched if $p<0.05$ and ≥3 significant features overlapped with known metabolites. Analysis parameters: mass tolerance 5 ppm; retention time in seconds; primary ions enforced; inclusion of pathways/metabolite sets with ≥3 entries.

Title: Supplementary Data 10. C18- features used for pathway enrichment analysis.
Description: C18- dataset used for pathway enrichment analysis in MetaboAnalyst (Mummichog v2.0). Significant features (one-way ANOVA, FDR-adjusted $p<0.05$) were used as input, with enrichment evaluated by permutation testing against all quality-filtered m/z features. Pathways were considered enriched if $p<0.05$ and ≥3 significant features overlapped with known metabolites. Analysis parameters: mass tolerance 5 ppm; retention time in seconds; primary ions enforced; inclusion of pathways/metabolite sets with ≥3 entries.
