# Supplementary material for: Clinically distinct metabotypes of pediatric MASLD identified through unsupervised clustering of NASH CRN data
Source: Nat Commun. 2026 Feb 24;17:3107. doi: 10.1038/s41467-026-69735-z (PMC13039967; doi:10.1038/s41467-026-69735-z)
Supplement: Supplementary file 4 — Reporting Summary [file 41467_2026_69735_MOESM4_ESM.pdf]

nature portfolio

Corresponding author(s): Helaina Huneault

Last updated by author(s): December 3 2025

Reporting Summary

Nature Portfolio wishes to improve the reproducibility of the work that we publish. This form provides structure for consistency and transparency in reporting. For further information on Nature Portfolio policies, see our Editorial Policies and the Editorial Policy Checklist.

Statistics

For all statistical analyses, confirm that the following items are present in the figure legend, table legend, main text, or Methods section.

n/a	Confirmed	
☐	☒	The exact sample size (*n*) for each experimental group/condition, given as a discrete number and unit of measurement
☐	☒	A statement on whether measurements were taken from distinct samples or whether the same sample was measured repeatedly
☐	☒	The statistical test(s) used AND whether they are one- or two-sided<br>*Only common tests should be described solely by name; describe more complex techniques in the Methods section.*
☐	☒	A description of all covariates tested
☐	☒	A description of any assumptions or corrections, such as tests of normality and adjustment for multiple comparisons
☐	☒	A full description of the statistical parameters including central tendency (e.g. means) or other basic estimates (e.g. regression coefficient) AND variation (e.g. standard deviation) or associated estimates of uncertainty (e.g. confidence intervals)
☐	☒	For null hypothesis testing, the test statistic (e.g. *F*, *t*, *r*) with confidence intervals, effect sizes, degrees of freedom and *P* value noted<br>*Give P values as exact values whenever suitable.*
☒	☐	For Bayesian analysis, information on the choice of priors and Markov chain Monte Carlo settings
☒	☐	For hierarchical and complex designs, identification of the appropriate level for tests and full reporting of outcomes
☒	☐	Estimates of effect sizes (e.g. Cohen's *d*, Pearson's *r*), indicating how they were calculated

Our web collection on statistics for biologists contains articles on many of the points above.

Software and code

Policy information about availability of computer code

Data collection	Serum samples were processed using commercial high-resolution LC-MS instruments (Orbitrap Fusion and HFQE, ThermoFisher Scientific) with instrument control software provided by the manufacturer. No custom software was used for primary data collection.
Data analysis	Data analysis was performed using RStudio (version 4.2.3) with several open-source packages including NbClust, factoextra, ggplot2, pheatmap, Hmisc, xMSanalyzer, and apLCMS. Pathway enrichment was conducted using MetaboAnalyst 6.0 with Mummichog v2.0, and integrative network analysis was performed using xMWAS (v0.552). Full analysis code and documentation are publicly available on GitHub: https://github.com/HHuneault/NASH_CRN_pediatric_metabotypes_R.

For manuscripts utilizing custom algorithms or software that are central to the research but not yet described in published literature, software must be made available to editors and reviewers. We strongly encourage code deposition in a community repository (e.g. GitHub). See the Nature Portfolio guidelines for submitting code & software for further information.

Data

Policy information about availability of data

All manuscripts must include a data availability statement. This statement should provide the following information, where applicable:

- Accession codes, unique identifiers, or web links for publicly available datasets
- A description of any restrictions on data availability
- For clinical datasets or third party data, please ensure that the statement adheres to our policy

The clinical and metabolomics datasets for the NAFLD Pediatric Database 2 (DB2) NASH CRN study are publicly accessible via the Human Health Exposure Analysis Resource (HHEAR) program website (Project #: 2017-1593) through the HHEAR Data Center and the Metabolomics Workbench (Study ID: ST001428).Clinical data from the Treatment of NAFLD in Children (TONIC) trial and the NAFLD Pediatric Database (DB1) NASH CRN studies are publicly available through the NIDDK Central Repository.Metabolomics data from these studies can be made available upon reasonable request to the investigators.

Research involving human participants, their data, or biological material

Policy information about studies with human participants or human data. See also policy information about sex, gender (identity/presentation), and sexual orientation and race, ethnicity and racism.

Reporting on sex and gender	Sex (male/female) was collected based on self-report and confirmed via clinical records. Gender was not separately assessed. The study included both male and female participants (73% male overall), and analyses stratified by sex were not performed due to the primary focus on metabolic phenotypes of MASLD. However, sex distribution across clusters was examined and visualized (see Figure S2), showing no significant separation by sex.
Reporting on race, ethnicity, or other socially relevant groupings	Ethnicity was self-reported by participants or caregivers and categorized as Hispanic or non-Hispanic. This variable was included in descriptive summaries and compared across clusters. Race was not used as a proxy for other variables. We acknowledge that sociocultural and environmental factors may influence metabolic phenotypes, but race/ethnicity were not specifically used in clustering analyses.
Population characteristics	Participants were children and adolescents aged 5 to 18 years with biopsy-confirmed MASLD. Covariates considered included age, sex, BMI percentile, liver enzymes (ALT, AST), lipid levels (LDL, VLDL, TG), waist circumference, uric acid, HOMA2-IR, and systolic blood pressure. These variables informed cluster identification and characterizations.
Recruitment	Participants were enrolled through three NASH CRN studies: TONIC (a randomized controlled trial), and the NAFLD Pediatric Databases (DB1 and DB2), both observational studies. Recruitment criteria included liver biopsy-confirmed MASLD and excluded participants with other liver diseases or confounding medical conditions. Enrollment was site-based across multiple U.S. pediatric centers, with potential bias toward Hispanic males noted and acknowledged as a limitation. Participant compensation followed the procedures outlined in each parent study.
Ethics oversight	The study protocol was approved by the Emory University Institutional Review Board and Children's Healthcare of Atlanta (STUDY00001715). Informed consent and assent were obtained from all participants and guardians.

Note that full information on the approval of the study protocol must also be provided in the manuscript.

Field-specific reporting

Please select the one below that is the best fit for your research. If you are not sure, read the appropriate sections before making your selection.

☒ Life sciences ☐ Behavioural & social sciences ☐ Ecological, evolutionary & environmental sciences

For a reference copy of the document with all sections, see nature.com/documents/nr-reporting-summary-flat.pdf

Life sciences study design

All studies must disclose on these points even when the disclosure is negative.

Sample size	The final sample size was 514 children with biopsy-proven MASLD from three NASH CRN studies. No formal sample size calculation was performed, as this was a secondary analysis using existing data from completed clinical studies. This cohort represents the largest publicly available pediatric MASLD dataset with paired clinical and metabolomics data and was considered sufficient for unsupervised clustering and metabolomic analysis, as larger sample sizes improve cluster stability and enhance robustness in high-dimensional metabolomics studies.
Data exclusions	A small number of participants (n=42) were excluded due to missing key clinical variables or being statistical outliers (>5 SD from the mean). One participant with HbA1c≥6.5% was excluded to ensure metabolic homogeneity. These exclusions were pre-defined and applied prior to clustering analysis. Outlier removal for metabolomics analysis was performed based on PCA and Hotelling's T^2 distribution (n=24).
Replication	This was a cross-sectional, observational analysis using data from three previously conducted NASH CRN studies. All findings are based on a single cohort and were not independently replicated. However, permutation testing and robustness checks were performed to ensure cluster stability and reproducibility of metabolomics findings. Validation in external cohorts is suggested as a next step.
Randomization	While participants were not randomized into experimental groups (as this was a secondary analysis of observational and trial data), serum

Randomization	samples for metabolomics analysis were randomized prior to LC-MS processing to minimize batch effects. Randomization was done independently for each batch, and pooled quality control samples were included throughout the runs to ensure data consistency.
Blinding	Investigators performing the clustering and metabolomics analyses were blinded to histological outcomes and ethnicity during initial data processing. Histology and ethnicity were used for cluster interpretation only after cluster formation.

Reporting for specific materials, systems and methods

We require information from authors about some types of materials, experimental systems and methods used in many studies. Here, indicate whether each material, system or method listed is relevant to your study. If you are not sure if a list item applies to your research, read the appropriate section before selecting a response.

Materials & experimental systems

n/a	Involved in the study
☒	☐ Antibodies
☒	☐ Eukaryotic cell lines
☒	☐ Palaeontology and archaeology
☒	☐ Animals and other organisms
☐	☒ Clinical data
☒	☐ Dual use research of concern
☒	☐ Plants

Methods

n/a	Involved in the study
☒	☐ ChIP-seq
☒	☐ Flow cytometry
☒	☐ MRI-based neuroimaging

Clinical data

Policy information about clinical studies

All manuscripts should comply with the ICMJE guidelines for publication of clinical research and a completed CONSORT checklist must be included with all submissions.

Clinical trial registration	TONIC trial: Registered at ClinicalTrials.gov (NCT00063635), NAFLD Pediatric Database 2 (DB2): Registered at ClinicalTrials.gov (NCT01061684), NAFLD Pediatric Database (DB1): Registered as an observational study at the NIDDK Central Repository (https://repository.niddk.nih.gov/study/166)
Study protocol	The full protocols for the TONIC trial (NCT00063635) and NAFLD Pediatric Database 2 (NCT01061684) are available on ClinicalTrials.gov. The protocol for the NAFLD Pediatric Database (DB1) is available via the NIDDK Central Repository: https://repository.niddk.nih.gov/study/166
Data collection	Data were collected from pediatric participants (ages 5–18 years) enrolled in three NASH Clinical Research Network (CRN) studies: TONIC (n=93), NAFLD Pediatric Database (DB1; n=23), and NAFLD Pediatric Database 2 (DB2; n=398). Recruitment and data collection occurred from 2004 to 2019 at multiple clinical sites across the U.S. Participants underwent liver biopsy, fasting blood draw, and standardized anthropometric and clinical assessments. Serum samples were stored at −80°C and later processed for high-resolution metabolomics at Emory University.
Outcomes	This was a retrospective cross-sectional study. The primary outcome was the identification of metabolic subtypes (metabotypes) of pediatric MASLD using unsupervised clustering of clinical data. Secondary outcomes included metabolic pathway differences and associations between metabolite features and fibrosis stage. Outcomes were assessed using fasting clinical biomarkers, histological data from liver biopsies, and serum metabolomics.

Plants

Seed stocks	*Report on the source of all seed stocks or other plant material used. If applicable, state the seed stock centre and catalogue number. If plant specimens were collected from the field, describe the collection location, date and sampling procedures.*
Novel plant genotypes	*Describe the methods by which all novel plant genotypes were produced. This includes those generated by transgenic approaches, gene editing, chemical/radiation-based mutagenesis and hybridization. For transgenic lines, describe the transformation method, the number of independent lines analyzed and the generation upon which experiments were performed. For gene-edited lines, describe the editor used, the endogenous sequence targeted for editing, the targeting guide RNA sequence (if applicable) and how the editor was applied.*
Authentication	*Describe any authentication procedures for each seed stock used or novel genotype generated. Describe any experiments used to assess the effect of a mutation and, where applicable, how potential secondary effects (e.g. second site T-DNA insertions, mosiacism, off-target gene editing) were examined.*

April 2023
